# G protein beta 3(*GNβ3*) C825T polymorphism and irritable bowel syndrome susceptibility: an updated meta-analysis based on eleven case-control studies

**DOI:** 10.18632/oncotarget.23449

**Published:** 2017-12-15

**Authors:** Dongbo Jiang, Dong Huang, Weiming Cai, Ting Li, Yan Wang, Huayan Chen, Tangming Guan, Xiaoli Ma

**Affiliations:** ^1^ Department of Pharmacy, Affiliated Hospital of Guangdong Medical University, Zhanjiang, Guangdong Province 524001, China; ^2^ Laboratory of Clinical Pharmacy, Guangdong Medical University, Zhanjiang, Guangdong Province 524001, China; ^3^ Department of Clinical Pharmacy, Guangdong Medical University, Zhanjiang, Guangdong Province 524001, China

**Keywords:** GNβ3, polymorphism, irritable bowel syndrome, case-control, meta-analysis

## Abstract

Several studies have reported an association between *GNβ3* C825T polymorphism and irritable bowel syndrome (IBS). However, the results remain inconclusive and controversial, particularly for the data derived from different ethnicities and IBS subtypes. Therefore, we performed an updated meta-analysis to evaluate this association. All eligible case-control studies that met the search criteria were retrieved from multiple databases, and eleven case-control studies were included for detailed evaluation. The pooled odds ratios (ORs) with 95% confidence intervals (95% CIs) were calculated to assess the strengths of the association between *GNβ3* C825T polymorphism and susceptibility to IBS and its subtypes. Our meta-analysis found no significantly associations of *GNβ3* C825T polymorphism with IBS risk in all populations. Whereas the C allele was demonstrated to be a decreased risk factor for constipation predominant IBS (IBS-C) in allele model. Additionally, the CC genotype was found to be associated with increased diarrhea predominant IBS (IBS-D) risk in recessive model. Subgroup analysis by ethnicity revealed that these associations held true for the Asian subpopulation. In conclusion, this meta-analysis suggests the C allele of *GNβ3* C825T might be associated with a decreased risk of IBS-C, and the CC genotype of *GNβ3* might be associated with increased IBS-D risk.

## INTRODUCTION

Irritable bowel syndrome (IBS) is the most prevalent gastrointestinal disorder characterized by abdominal discomfort, pain, and altered defecation patterns; it may considerably reduce patients’ quality of life and work productivity, which affects more than 7 percent of people all around the world [1, 2]. According to the recurrent symptoms, IBS patients can experience constipation (IBS-C), diarrhea (IBS-D), mixture of diarrhea and constipation IBS (IBS-M) and un-subtyped IBS [3, 4]. IBS is a multifactorial disorder that is associated with biological and psychosocial factors [5–7]. Although genetic predisposition has been demonstrated in classical family and twin studies, unequivocal susceptibility genes have yet to be identified [2, 8]. Recently, several genetic association studies identified the guanine nucleotide binding protein (G-protein) *β3* subunit gene (*GNβ3*) C825T polymorphism as being significantly associated with IBS [9–12].

G-protein, consisting of an α, β, and γ subunit, is an intracellular second messenger signalling protein linked to a transmembrane receptor [13, 14]. The C825T polymorphism of *GNβ3* (rs5443) is associated with alternative splicing of the gene and its protein activity, and an increased intracellular signal transduction compared with unmodified Gβ3 protein [15, 16]. This single nucleotide polymorphism (SNP) has been reported to be associated with depression [17], Alzheimer's disease (AD) [18], hypertension [19], obesity [20], Insulin-mediated venodilation [21], Vasculogenic erectile dysfunction (VED) [22], and functional dyspepsia [23]. Recently, the association between the *GNβ3 C825T* polymorphism and the risk of IBS has been intensively investigated. However, the community is still unable to reach a consensus, particularly regarding the data from different ethnicities and IBS subtypes [9–12, 24–30]. To date, only one meta-analyses has reported on the relationship between *GNβ3* C825T polymorphism and susceptibility to IBS [31]. However, that meta-analysis only included seven studies, and missed many published articles [27–30]. Moreover, the previous meta-analysis did not analyze the association between *GNβ3* C825T polymorphism and different IBS subtypes with respect to ethnicity. Therefore, we conducted an updated meta-analysis including all published studies accompanied with ethnic subgroup analyses and IBS subtype analyses to clarify whether *GNβ3* C825T was associated with the development of IBS and its subtypes.

## RESULTS

### Characteristics of studies 26-9-11

**Figure 1:**
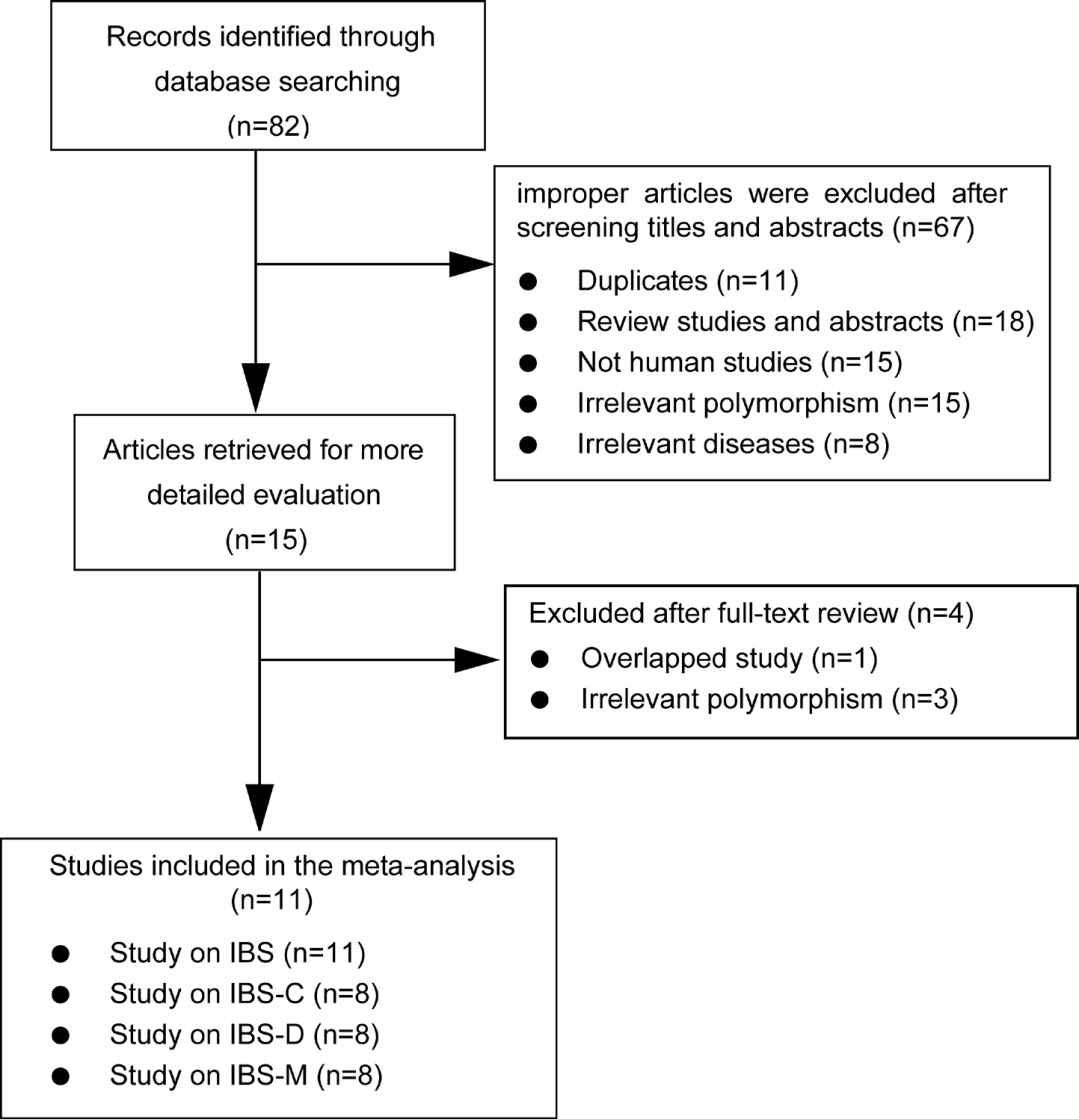
Flow diagram of selection of eligible studies

As showed in Figure 1, eleven studies involving 1,422 cases and 2,073 controls were ultimately included in the present meta-analysis, and eight of them [10, 12, 24, 25, 27–30] specifically investigated the association between *GNβ3* C825T polymorphism and different IBS subtypes (including the IBS-C, IBS-D and IBS-M) risk. The main characteristics of the included articles were summarized in Table 1. All of these included articles were case-control studies, of which nine in a hospital-based design [10–12, 25–30], two in a population-based design [9, 24]. Among these studies, six were on Caucasians [9, 11, 24, 25, 27, 28] and the other five were on Asians [10, 12, 26, 29, 30]. Additionally, all of the included studies were of high quality, as indicated by the Newcastle-Ottawa scale (NOS) scores of each study being above 6 points, and the genotype distributions in all of the controls were consistent with Hardy-Weinberg equilibrium (HWE), except one [10]. Studies with controls not in HWE were also considered for the meta-analysis, but they were excluded in the sensitivity analysis.

**Table 1:** Characteristics of studies included in the meta-analysis

Study	Country	Ethnicity	Design	Gender (M:F [n])	Genotype distribution (case/control)			Genotyping Method	Diagnosis criteria	HWE (P)	NOS
				(case/control)	CC	CT	TT				
Andresen V 2006	United States	Caucasian (95.1%)	PB	31:183 / 40:112	111/ 77	87/ 62	16/ 13	Direct Sequencing	Rome II	0.92	9
Camilleri M 2008	United States	Caucasian	HB	3:119/ 0:39	57/ 19	53/ 16	12/ 4	Direct Sequencing	Rome II	0.82	7
de Vries DR 2009	Netherlands	Caucasian	PB	66:70 / 120:253	60/ 199	68/ 138	8/ 36	molecular beacon assay	Rome II	0.10	8
Kim HG 2012	South Korea	Asian	HB	25:35 / 167:267	16/ 112	31/ 215	13/ 107	TaqMan Assay	Rome III	0.85	7
Lee HJ 2010	South Korea	Asian	HB	58:36 / 44:44	13/ 16	49/ 56	32/ 16	PCR and RFLP	Rome III	0.01	7
Markoutsaki T 2011	Greece	Caucasian	HB	30:94/ 96:142	37/ 120	65/ 97	22/ 21	PCR and RFLP	Rome III	0.82	7
Park CS 2012	South Korea	Asian	HB	32:40/ 81:67	27/ 35	28/ 79	17/ 34	SNaPShot	Rome III	0.41	7
Saito YA 2007	United States	Caucasian (> 96%)	HB	9:41/ 10:43	25/ 24	19/ 22	6/ 7	Thermo Electron Hybaid MBS thermal cycler	Rome II	0.59	7
Saito YA 2012	United States	Caucasian (> 97%)	HB	65:320/ 84:178	122/ 114	117/ 109	28/ 28	Thermo Electron Hybaid MBS thermal cycler	Rome II	0.80	7
Yoon Jin Choi 2014	South Korea	Asian	HB	38:61 / 85:86	26/ 40	46 / 81	27 / 50	TaqMan Assay	Rome III	0.52	8
Yuezhi Wang 2014	China	Asian	HB	56:10/ 89:26	50/ 92	13/ 21	3/ 2	Real-Time PCR	Rome III	0.54	7

Abbreviations: HB, hospital-based study; PB, population based; RFLP, restriction fragment length polymorphism analyses

### Power analysis

Before implementation of this meta-analysis, statistical power was assessed with the assumptions:α err prob = 0.05, OR = 1.25 (corresponding to a “weak to moderate” gene effect) for the SNP, and minor allele frequencies(MAF) of *GNβ3* C825T(rs5443, C/T) was estimated from the 1000 Genomes. The present samples indicated that 100% power to evaluate the association between this polymorphism and IBS. And the power to evaluate the associations between this polymorphism and IBS subtypes (IBS-C, IBS-D and IBS-M) were 88.1%, 98.3% and 93.9%, respectively. The power analysis indicated that these recruited samples could provide sufficient power in identifying the association between *GNβ3* C825T(rs5443, C/T) polymorphism and IBS and its subtypes.

### Quantitative synthesis

#### *GNβ3* C825T and IBS risk

**Figure 2:**
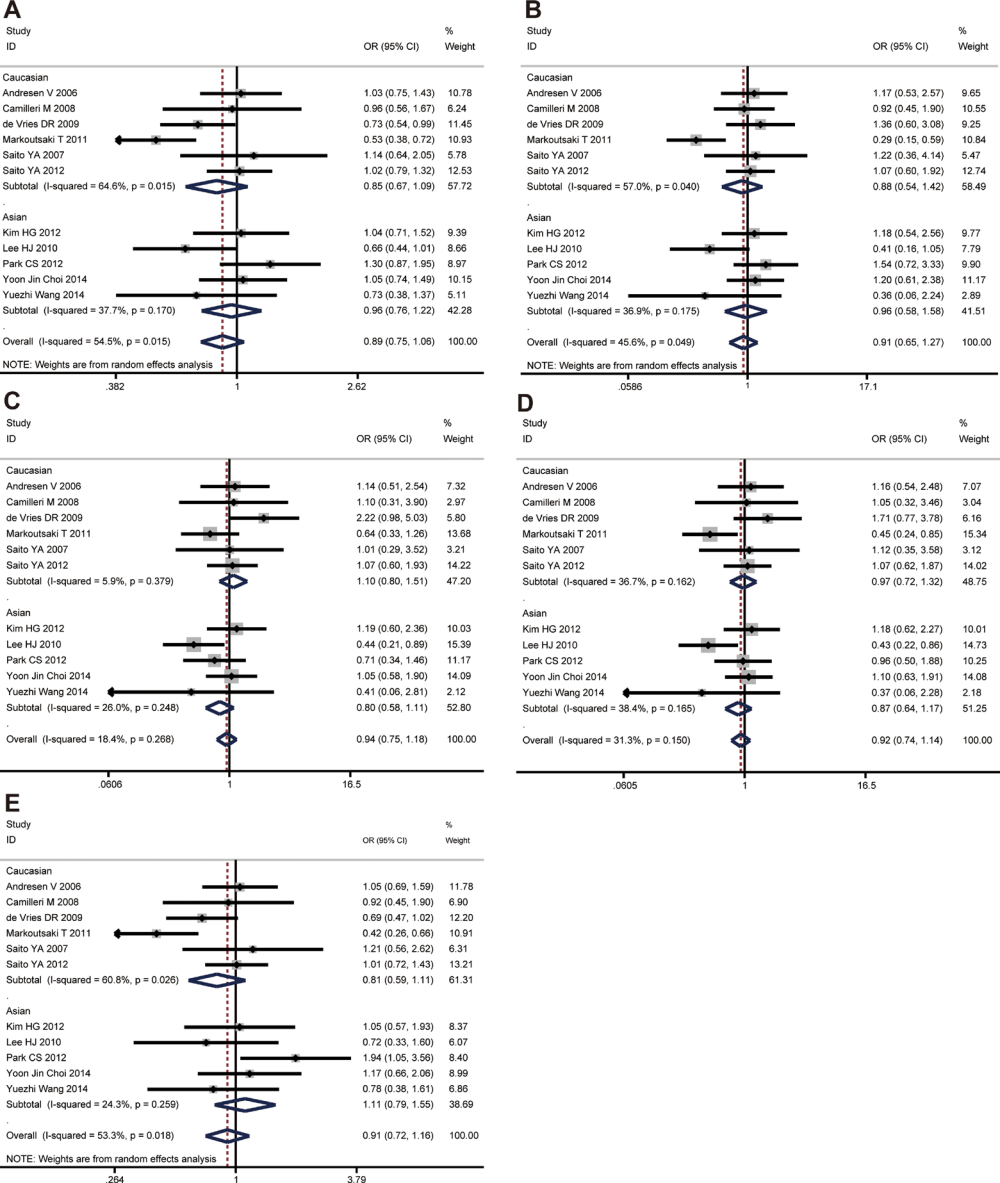
The associations of *GNβ3* C825T with IBS in different genetic models (**A**) Allele model (C vs. T). (**B**) Codominant model (CC vs. TT). (**C**) Codominant model (CT vs. TT). (**D**) Dominant model (CC + CT vs. TT). (**E**) Recessive model (CC vs. TT + CT).

Overall, no significant association between *GNβ3* C825T polymorphism and risk of IBS was observed under all genetic models (C vs. T, *P* = 0.194; CC vs. TT, *P* = 0.564; CT vs. TT, *P* = 0.594; CC + CT vs. TT, *P* = 0.430; CC vs. CT+TT, *P* = 0.462) (Figure 2). In the subgroup analyses by ethnicity, significant associations were not found in any genetic model for Asians and Caucasians. All the results are listed in Table 2.

**Table 2:** Summary of meta-analysis for the association of GNβ3 C825T polymorphism with IBS and its subtypes

Genetic models	Stratifications	Number of studies	OR (95%CI)	*P* value	Heterogeneity	
					*I^2^*	*P_H_*
**IBS**						
**C vs. T**	Overall	11	0.893 [0.752, 1.060]	0.194	54.5%	0.015
	Asians	5	0.959 [0.756, 1.216]	0.729	37.7%	0.170
	Caucasian	6	0.852 [0.666, 1.091]	0.205	64.6%	0.015
**CC vs. TT**	Overall	11	0.906 [0.649, 1.266]	0.564	45.6%	0.049
	Asians	5	0.960 [0.583, 1.580]	0.872	36.9%	0.175
	Caucasian	6	0.879 [0.543, 1.421]	0.599	57.0%	0.040
**CT vs. TT**	Overall	11	0.940 [0.748, 1.180]	0.594	18.4%	0.268
	Asians	5	0.800 [0.578, 1.107]	0.179	26.0%	0.248
	Caucasian	6	1.096 [0.796, 1.509]	0.574	5.9%	0.379
**CC+CT vs. TT**	Overall	11	0.917 [0.739, 1.137]	0.430	31.3%	0.150
	Asians	5	0.866 [0.639, 1.175]	0.356	38.4%	0.165
	Caucasian	6	0.970 [0.715, 1.316]	0.845	36.7%	0.162
**CC vs. CT+TT**	Overall	11	0.914 [0.718, 1.162]	0.462	53.3%	0.018
	Asians	5	1.110 [0.794, 1.552]	0.542	24.3%	0.259
	Caucasian	6	0.814 [0.595, 1.113]	0.197	60.8%	0.026
**IBS-C**						
**C vs. T**	Overall	8	0.788 [0.622, 0.997]	**0.048**	24%	0.238
	Asians	4	0.520 [0.329, 0.821]	**0.005**	0%	0.503
	Caucasian	4	0.926 [0.701,1.225]	0.592	0%	0.461
**CC vs. TT**	Overall	8	0.622 [0.370, 1.046]	0.073	0%	0.532
	Asians	4	0.258 [0.094, 0.707]	**0.008**	0%	0.736
	Caucasian	4	0.974 [0.506, 1.877]	0.938	0%	0.827
**CT vs. TT**	Overall	8	0.709 [0.449, 1.120]	0.140	0%	0.552
	Asians	4	0.431 [0.221, 0.842]	**0.014**	0%	0.749
	Caucasian	4	1.094 [0.571, 2.093]	0.787	0%	0.839
**CC+CT vs. TT**	Overall	8	0.653 [0.422, 1.011]	0.056	6.4%	0.381
	Asians	4	0.378 [0.200, 0.714]	**0.003**	0%	0.652
	Caucasian	4	1.035 [0.556, 1.929]	0.913	0%	0.848
**CC vs. CT+TT**	Overall	8	0.835 [0.597, 1.168]	0.292	0%	0.731
	Asians	4	0.513 [0.218, 1.208]	0.126	0%	0.668
	Caucasian	4	0.927 [0.641, 1.340]	0.686	0%	0.621
**IBS-D**						
**C vs. T**	Overall	8	1.162 [0.977, 1.381]	0.089	30.9%	0.181
	Asians	4	1.316 [1.019, 1.700]	**0.035**	55.8%	0.079
	Caucasian	4	1.046 [0.827, 1.321]	0.709	0%	0.635
**CC vs. TT**	Overall	8	1.246 [0.866, 1.793]	0.236	14.8%	0.314
	Asians	4	1.609 [0.963, 2.690]	0.069	43.9%	0.148
	Caucasian	4	0.957 [0.571, 1.603]	0.867	0%	0.802
**CT vs. TT**	Overall	8	0.843 [0.592, 1.201]	0.345	0%	0.822
	Asians	4	0.796 [0.493, 1.287]	0.352	9.1%	0.348
	Caucasian	4	0.902 [0.543, 1.525]	0.701	0%	0.979
**CC+CT vs. TT**	Overall	8	0.996 [0.716, 1.384]	0.980	0%	0.542
	Asians	4	1.048 [0.674, 1.630]	0.836	43.6%	0.150
	Caucasian	4	0.934 [0.570, 1.529]	0.785	0%	0.901
**CC vs. CT+TT**	Overall	8	1.268 [1.000, 1.608]	**0.050**	27.8%	0.206
	Asians	4	1.688 [1.157, 2.463]	**0.007**	29.2%	0.237
	Caucasian	4	1.054 [0.777, 1.432]	0.735	0%	0.644
**IBS-M**						
**C vs. T**	Overall	8	0.789 [0.576, 1.080]	0.139	51.5%	0.044
	Asians	4	0.672 [0.403, 1.121]	0.128	50.1%	0.111
	Caucasian	4	0.926 [0.660, 1.299]	0.655	34.5%	0.205
**CC vs. TT**	Overall	8	0.798 [0.402, 1.587]	0.521	38.1%	0.126
	Asians	4	0.464 [0.126, 1.713]	0.249	53.1%	0.094
	Caucasian	4	1.253 [0.678, 2.316]	0.472	0.0%	0.513
**CT vs. TT**	Overall	8	0.994 [0.662, 1.495]	0.979	0%	0.614
	Asians	4	0.755 [0.431, 1.325]	0.327	0%	0.622
	Caucasian	4	1.337 [0.732, 2.440]	0.345	0%	0.540
**CC+CT vs. TT**	Overall	8	0.941 [0.639, 1.387]	0.760	11.6%	0.340
	Asians	4	0.689 [0.404, 1.174]	0.170	9.5%	0.346
	Caucasian	4	1.306 [0.736, 2.318]	0.361	0.0%	0.529
**CC vs. CT+TT**	Overall	8	0.788 [0.500, 1.240]	0.303	45.2%	0.078
	Asians	4	0.622 [0.216, 1.789]	0.378	61.5%	0.050
	Caucasian	4	0.935 [0.623, 1.403]	0.746	21.6%	0.281

#### *GNβ3* C825T and IBS-C risk

**Figure 3:**
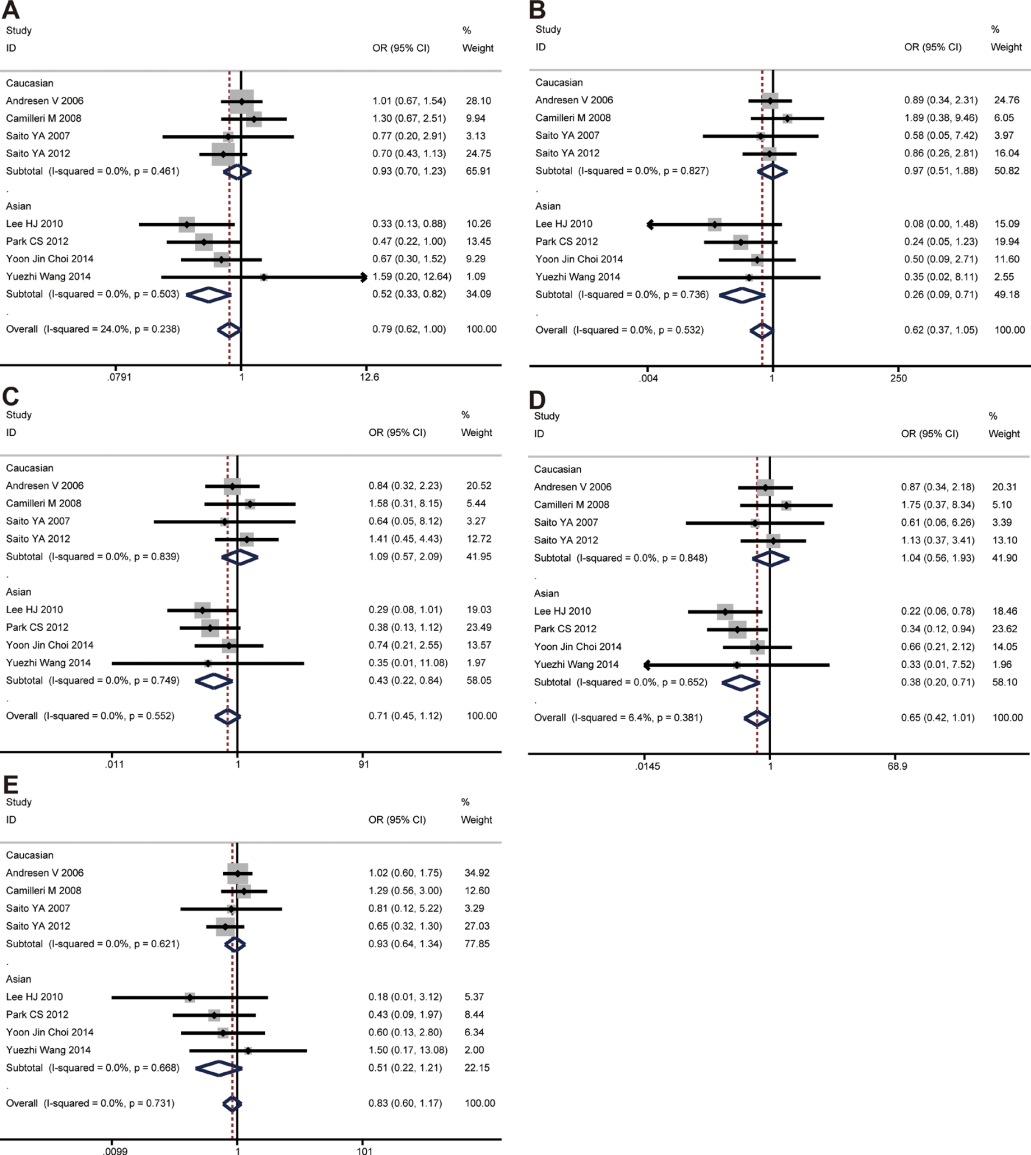
The associations of *GNβ3* C825T with IBS-C in different genetic models (**A**) Allele model (C vs. T). (**B**) Codominant model (CC vs. TT). (**C**) Codominant model (CT vs. TT). (**D**) Dominant model (CC + CT vs. TT). (**E**) Recessive model (CC vs. TT + CT).

The C allele of *GNβ3* C825T was found to be significantly associated with a decreased risk of IBS-C in allele model (C vs. T, OR = 0.788, 95% CI: 0.622-0.997, *P* = 0.048), while no evidence of significance was identified in other genetic models (CC vs. TT, *P* = 0.073; CT vs. TT, *P* = 0.140; CC+CT vs. TT, *P* = 0.056; CC vs. CT+TT, *P* = 0.292). In the subgroup analyses by ethnicity, the significant association was found among Asians under all genetic models (C vs. T, OR = 0.520, 95% CI: 0.329-0.821, *P* = 0.005; CC vs. TT, OR = 0.258, 95% CI: 0.094-0.707, *P* = 0.008; CT vs. TT, OR = 0.431, 95% CI: 0.221-0.842, *P* = 0.014; CC+CT vs. TT, OR = 0.378, 95% CI: 0.200-0.714, *P* = 0.003) except for the recessive model (CC vs. CT+TT, *P* = 0.126) (Figure 3). However, there was no significant association between this polymorphism and IBS-C development in the Caucasian population under any genetic model. All the results are listed in Table 2.

#### *GNβ3* C825T and IBS-D risk

**Figure 4:**
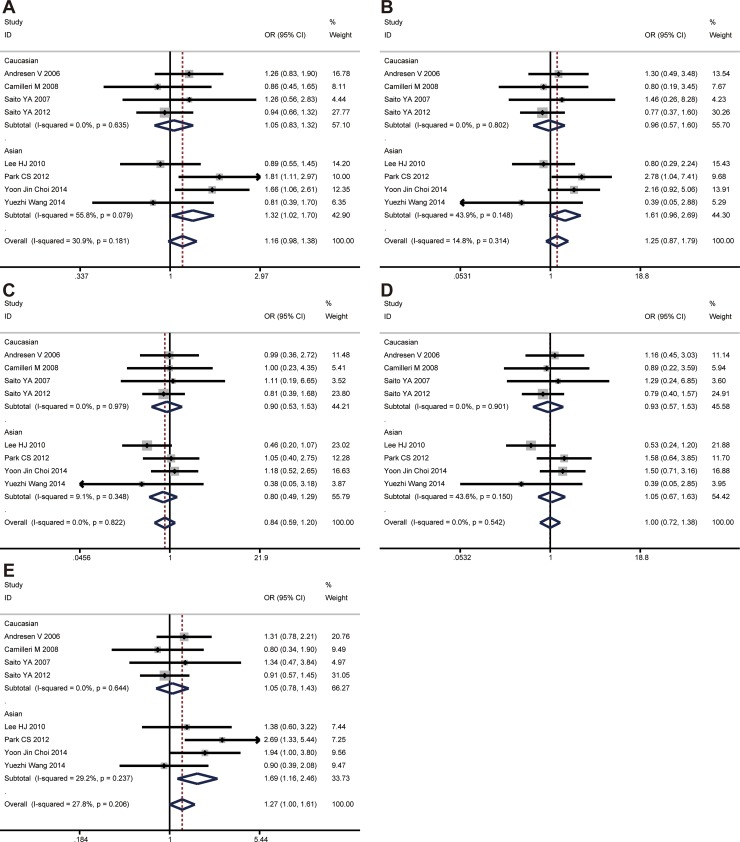
The associations of *GNβ3* C825T with IBS-D in different genetic models (**A**) Allele model (C vs. T). (**B**) Codominant model (CC vs. TT). (**C**) Codominant model (CT vs. TT). (**D**) Dominant model (CC + CT vs. TT). (**E**) Recessive model (CC vs. TT + CT).

The CC genotype of *GNβ3* C825T was found to be significantly associated with an increased risk of IBS-D in recessive model (CC vs. CT + TT, OR = 1.268, 95% CI: 1.000-1.608, *P* = 0.050), while no evidence of significance was identified in other genetic models (C vs. T, *P* = 0.089; CC vs. TT, *P* = 0.236; CT vs. TT, *P* = 0.345; CC + CT vs. TT, *P* = 0.980). In the subgroup analyses by ethnicity, the significant association was found among Asians under allele model (C vs. T, OR = 1.316, 95% CI: 1.019–1.700, *P* = 0.035) and recessive model (CC vs. CT+TT, OR = 1.688, 95% CI: 1.157–2.463, *P* = 0.007), while no evidence of significance was identified in other two genetic models (Figure 4). However, there was no significant association between this polymorphism and IBS-D development in the Caucasian population under any genetic model. All the results are listed in Table 2.

#### *GNβ3* C825T and IBS-M risk

No significant association was found between *GNβ3* C825T polymorphism and IBS-M in the overall population. In subgroup analysis, there was no significant association between this polymorphism and IBS-M development in the Caucasian and Asian population. All the results are listed in Table 2.

### Sensitivity analysis

**Figure 5:**
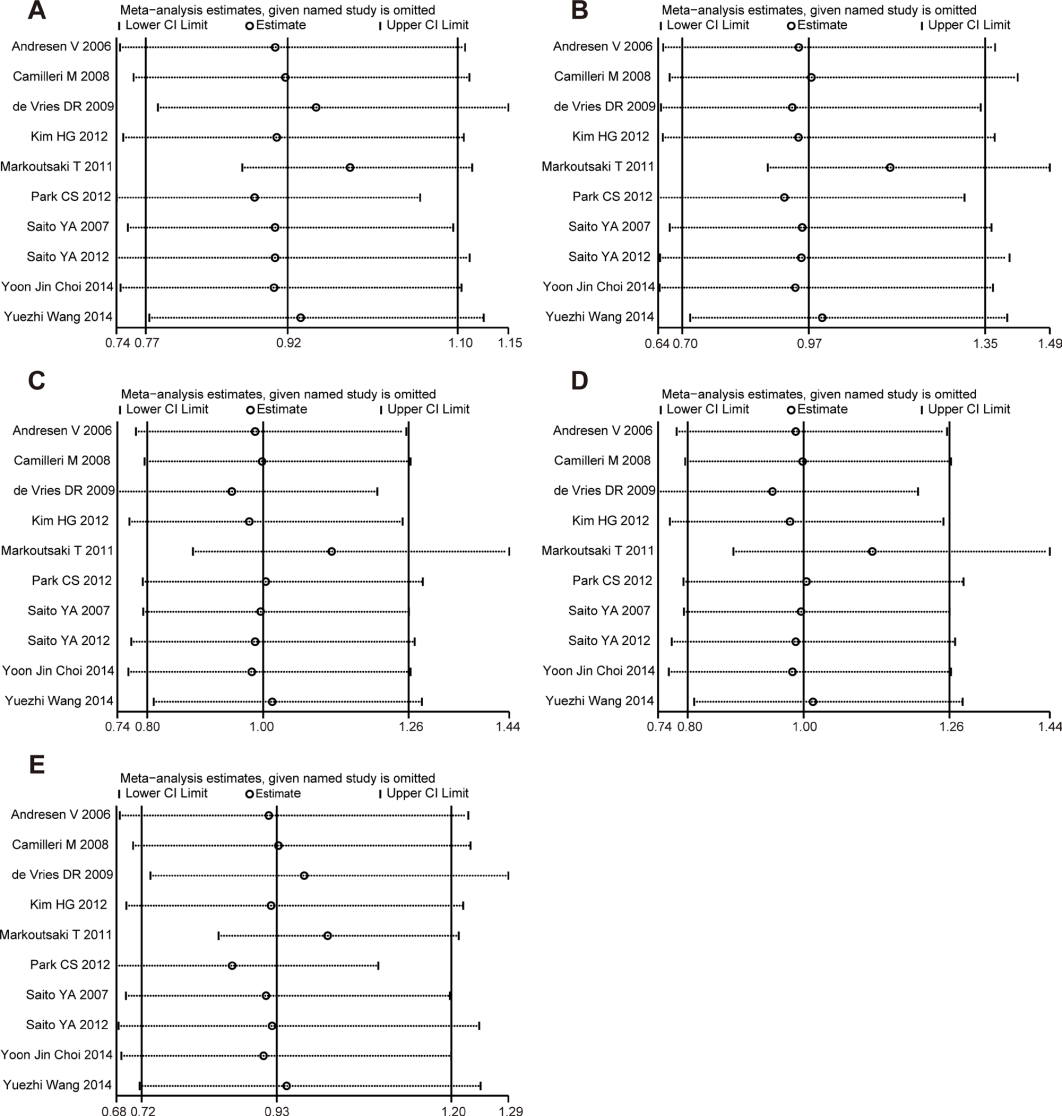
Sensitivity analysis of the association of *GNβ3* C825T and IBS in the different genetic models (**A**) Allele model (C vs. T). (**B**) Codominant model (CC vs. TT). (**C**) Codominant model (CT vs. TT). (**D**) Dominant model (CC + CT vs. TT). (**E**) Recessive model (CC vs. TT + CT).

Sensitivity analysis of the summary odds ratio coefficients on the relationship of the SNP and the risk of IBS is computed by omitting each study in turn. The corresponding pooled ORs were not significantly altered after excluding each eligible study at a time (Figure 5).

### Publication bias

No evidence of publication bias was detected regarding the ORs of the SNP in this study by either Begg's or Egger's test (Table 3).

**Table 3:** Publication bias tests for association of the GNβ3 C825T polymorphism with IBS

Comparisons	Egger test			Begg test
	Coefficient	*P* value	95% CI	*P* value
**GNβ3 C825T**				
C vs. T	0.48	0.80	(–3.70, 4.67 )	0.76
CC vs. TT	–0.96	0.60	(–4.92, 3.01 )	0.64
CT vs. TT	–0.22	0.87	(–3.02, 2.59 )	0.76
CC+CT vs. TT	–0.34	0.80	(–3.39, 2.70 )	0.64
CC vs. CT+TT	0.93	0.59	(–2.75, 4.64 )	0.64

## DISCUSSION

Recently, several genetic association studies identified a novel association between *GNβ3 C825T* polymorphism and IBS. In Asia, Lee et al. [10] demonstrated that *GNβ3* 825T allele is associated with IBS in Koreans, especially among IBS with constipation. Likewise, Park et al. [12] found that the TT genotype of *GNβ3* C825T is common in IBS-C while the CC genotype is common in IBS-D. In Europe, Markoutsaki T [11] found that TT genotype and T allele of *GNβ3* are significantly associated with IBS predisposition in Greeks. Besides, in a Netherlands study CT genotype of *GNβ3* showed significant association with IBS [9]. However, other studies [24–30] revealed that the *GNβ3* C825T polymorphism may be not associated with the development of IBS or its subtypes. Generally, this disparity might be partly due to ethnic differences or to the limited numbers of subjects involved in the studies. To derive a more precise estimation of this association, we performed a meta-analysis to clarify the associations between the *GNβ3* C825T mutation and the presence of IBS and its subtypes. Eleven case-control studies [9–12, 24–30] with a total of 1,422 IBS patients and 2,073 healthy controls were included in our meta-analysis, which was sufficiently powered to detect IBS susceptibility associated with *GNβ3* C825T gene polymorphism.

In the present study, we used four models to estimate the relationship between *GNB3* C825T polymorphism and IBS and its subtypes. The C allele of *GNβ3* C825T was demonstrated to be significantly associated with a decreased risk of IBS-C in allele model. While the results of the recessive model supported CC genotype of *GNβ3* could increase the risk of IBS-D. Additionally, subgroup analyses by ethnicity indicated that the SNP of *GNβ3* C825T was only significantly associated with a decreased risk of IBS-C in Asian population, while the CC genotype was only associated with increased IBS-D risk in the Asian population. Besides that, neither the overall results with the whole population nor the subgroup analysis by Asian and Caucasian ethnicity indicated the associations between *GNβ3* C825T polymorphism and the development of IBS and IBS-M. Obviously, our results are not consistent with some previous studies [9–12] that *GNβ3* C825T was associated with IBS. A possible explanation for this phenomenon is that the previous single studies of IBS had small samples size, and thus the significance of current work may not be justified; thus, further studies are needed to clarify the effects of this SNP on the development of IBS and its subtypes. In addition, the differential allele frequencies of the SNP exerted disproportionate levels of influence on the IBS risks in different populations. For example, the minor allele frequencies (MAF) of the SNP *GNβ3* C825T (rs5443) is 0.50 in the East Asian population (EAS), whereas the MAF is 0.31 in the European population (EUR) and the MAF is 0.38 in the Ad Mixed American population (AMR) based on the data from the 1000 G. In accordance with our partial findings, a previous meta-analysis performed by Pan ZG et al. [31] found that no associations of GNB3 C825T polymorphism with IBS risk either in Asian population or Caucasian population. However, that previous meta-analysis based on seven studies still had some differences from our results, and suggested that no significant associations between *GNβ3* C825T polymorphism and 3 IBS subtypes (IBS-C, IBS-D and IBS-M). The major reason of this discrepancy is that this previous meta-analysis did not include all the published articles and did not assess the association between this polymorphism and IBS subtypes with respect to ethnicity. In contrast, our meta-analysis included all the eleven relevant published studies with a larger sample size of cases and controls, which gave a greater statistical power to evaluate the association than the previous study. Additionally, our study included four studies in Asian group and four studies in Caucasian group to evaluate the association between *GNβ3* C825T polymorphism and IBS subtypes, which gave a more detailed analysis to assay the association and showed a more reliable result.

To the best of our knowledge, this is the first meta-analysis to explore the relationships between *GNβ3* C825T gene polymorphism and IBS and its subtypes with respect to ethnicity. The genotype distributions in all of the controls were consistent with HWE, except one for one study reported by Lee et al. [10]. However, the association was not significant change when excluded the study. The NOS results indicated that the included studies were credible. Moreover, sensitivity analysis did not significantly alter in overall and subgroup results under all genetic models. In addition, no evidence of publication bias was identified by either Begg's or Egger's tests. Taken together, the outcomes of our meta-analysis are relatively reliable and stable.

Nevertheless, there were some limitations in the current study. First, only articles in English and Chinese language were included; thus, studies written in other languages were neglected. Second, although we performed a systematic searching strategy to identify eligible studies, there was still probability that few studies so called “grey literatures” were not included. Third, due to the limited data, we did not carry out subgroup analysis to other factors, which may participate in the progression of IBS, such as age, infection, social psychology, and other living habits. Finally, IBS is a common gastrointestinal disorder in the human population; particularly in females (two thirds of patients are female). However, none of the original studies accounted for gene-gender interactions. Further studies are needed to clarify whether gender-related differences affected the polymorphism of *GNβ3* C825T and subsequent IBS.

In summary, this meta-analysis suggested that the C allele of *GNβ3* C825T may be associated with a decreased risk of IBS-C, while the CC genotype of *GNβ3* may be associated with increased IBS-D risk. However, due to the above-mentioned limitations, a well-designed large-scale study that includes ethnicities, IBS subtypes and psychosocial factors is required to confirm the findings of the current meta-analysis.

## MATERIALS AND METHODS

### Search strategy and selection criteria

According to the Preferred Reporting Items for Systematic Reviews and Meta-Analyses (PRISMA) statement [32], we searched the related literature of the electronic records of the PubMed, Embase, Science Direct, Chinese National Knowledge Infrastructure (CNKI) and WANFANG databases prior to April 2017. The search terms included the following key words: (“Irritable bowel syndrome” *or* “IBS”) AND (“polymorphism” *or* “allele” *or* “gene” *or* “mutation” *or* “variant”) AND (“G protein beta3” or “G protein *β3*” or “*GNβ3*”). Furthermore, the references of all retrieved articles were also checked by hand to identify additional potential studies. The languages were limited to English and Chinese. We inclusion all studies that (1) evaluated the association between *GNβ3* polymorphism and the risk of IBS; (2) used a case control design; (3) provided sufficient data of allele and genotype frequencies of SNPs or required information could be calculated; and (4) if serial studies on the same population were published, only the most recent or the largest research study was included. Additionally, we excluded reviews, abstracts, and redundant and animal studies.

### Data extraction

Two independent investigators extracted relevant data from all included studies on the basis of the inclusion criteria, and a third investigator verified them. The following information was extracted from all of enrolled studies: the surname of the first author, publication year, country of origin, ethnicity, sample size, genotyping method, and the *GNβ3* genotype distributions and alleles in the case and control groups.

### Quality assessment

The quality of included studies were assessed by two investigators independently on the basis of Newcastle-Ottawa Scale (NOS) [33], which based on three aspects: selection, comparability and exposure. Studies with a score of 5 points or higher were considered to be of high quality.

### Statistical analysis

The HWE of the genotype distributions in the controls of the include studies were tested by the Chi-square test, *P* < 0.05 was considered statistically significant. Studies with the controls not in HWE were subjected to a sensitivity analysis [34]. The power analysis was calculated by using the Power and Sample Size Program software [35]. The associations of the *GNβ3* polymorphism with the risk of IBS was assessed by the pooled ORs with the corresponding 95% CIs under the following genetic models: allele model (C vs. T), codominant model (CC vs. TT and CT vs. TT), dominant model (CC + CT vs. TT), and recessive model (CC vs. CT + TT). The heterogeneity between studies was determined by the Cochrane's *Q*-statistic test [36], and the inconsistency was quantified with the *I*^2^ statistic. When *I*^2^ > 50% or *P*_Q_ ≤ 0.1, which suggest substantial heterogeneity, a random-effects model (DerSimonian-Laird method) [37] was used; otherwise, the fixed-effects model (Mantel-Haenszel method) [32] was applied. Sensitivity analysis was conducted by sequentially omitting each study to evaluate the stability of statistical results. Furthermore, Begg's funnel plot and Egger's test [38] was used to evaluate the potential publication bias (*P* < 0.05 was considered statistically significant). All analyses were conducted using the STATA 12.0 software packages.

## Abbreviations

IBS: Irritable bowel syndrome
IBS-C: constipation predominant IBS
IBS-D: diarrhea predominant IBS
IBS-M: mixture of diarrhea and constipation IBS
ORs: odds ratios
95% CIs: 95% confidence intervals
G-protein: Guanine nucleotide binding protein
*GNβ3*: G-protein *β3* subunit gene
SNP: single nucleotide polymorphism
HWE: Hardy-Weinberg equilibrium
NOS: Newcastle- Ottawa Scale
MAF: minor allele frequencies
EAS: East Asian population
AMR: Ad Mixed American population
EUR: European population
PRISMA: Preferred Reporting Items for Systematic Reviews and Meta-Analyses
CNKI: Chinese National Knowledge Infrastructure.
